# Immunosurveillance against cancer-associated hyperploidy

**DOI:** 10.18632/oncotarget.753

**Published:** 2012-11-20

**Authors:** Laura Senovilla, Lorenzo Galluzzi, Guillermo Mariño, Ilio Vitale, Maria Castedo, Guido Kroemer

**Affiliations:** INSERM, U848, Villejuif, France; Institut Gustave Roussy, Villejuif, France; Université Paris Sud/Paris XI, Le Kremlin Bicétre, France; Institut Gustave Roussy, Villejuif, France; Université Paris Descartes/Paris V, Sorbonne Paris Cité, Paris, France; INSERM, U848, Villejuif, France; Institut Gustave Roussy, Villejuif, France; Université Paris Sud/Paris XI, Le Kremlin Bicêtre, France; 1INSERM, U848, Villejuif, France; 2Institut Gustave Roussy, Villejuif, France; Université Paris Sud/Paris XI, Le Kremlin Bicêtre, France; INSERM, U848, Villejuif, France; Institut Gustave Roussy, Villejuif, France; Université Paris Sud/Paris XI, Le Kremlin Bicêtre, France; INSERM, U848, Villejuif, France; Université Paris Descartes/Paris V, Sorbonne Paris Cité, Paris, France; Metabolomics Platform, Institut Gustave Roussy, Villejuif, France; Centre de Recherche des Cordeliers, Paris, France; Pôle de Biologie, Hôpital Européen Georges Pompidou, AP-HP, Paris, France

At odds with long-standing convictions, it is now clear that tumors are not immunologically silent entities. We have recently demonstrated that the immune system can selectively detect and eliminate hyperploid (pre-)malignant cells, thus delineating a novel mechanism of immunosurveillance against tumorigenesis.

Malignant cells of different histological origin share a set of common features, including an impressive, growth factor-independent proliferative potential as well as an increased resistance to potentially lethal stimuli. At least in part, such characteristics stem from mutations in the genome of (pre-)neoplastic cells that cause either the loss-of-function of oncosuppressor genes and/or the (hyper)activation of proto-oncogenes [1]. Along with tumor progression, newly-formed malignant cells continue to accumulate genetic defects that promote a state of increased aggressiveness. In some cases, (pre-)malignant cells undergo gross chromosomal rearrangements, conferring them additional proliferative advantages [2]. Although the exact sequence and timing of the molecular events that underpin malignant transformation remain largely obscure, an elevated degree of genomic instability is often associated with enhanced tumor growth and aggressiveness. In many instances, genomic instability develops along with aneuploidy, a non-diploid state that frequently derives from tetraploidy, via multipolar mitosis, asymmetric cell division and/or chromosome losses [3]. In line with this notion, several cell-intrinsic mechanisms are in place to prevent the survival, proliferation or replication of illicitly generated tetraploid and aneuploid cells, including multiple cell cycle checkpoints and mitotic catastrophe [4].

**Figure 1:**
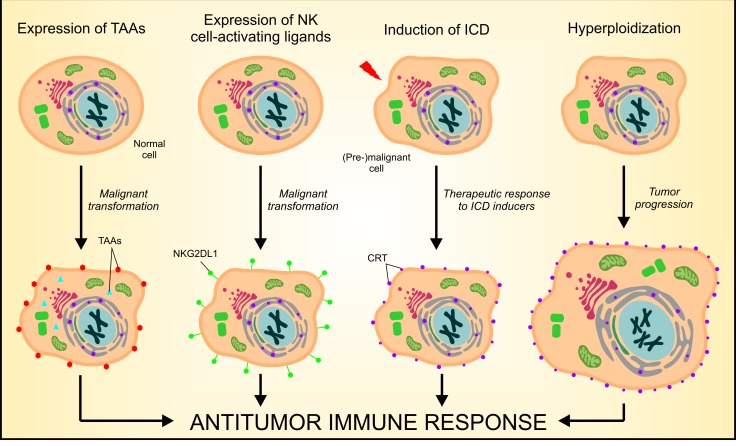
Mechanisms of cancer cell recognition by the immune system There are at least four distinct mechanisms whereby the immune system can detect - and potentially eliminate - (pre-)neoplastic cells. First, cancer cells often express tumor-associated antigens (TAAs) that - at least theoretically - can elicit anticancer immune responses. Second, (pre-)malignant cells often express increased amount of natural killer (NK)-cell activating receptor ligands (*e.g.*, NKG2DL1) on their surface, hence promoting the activation of innate immunosurveillance mechanisms. Third, some antineoplastic agents, including anthracyclines and oxaliplatin, are capable of eliciting immunogenic cell death (ICD), ultimately leading to the activation of cognate tumor-specific immune responses. Forth, the immune system can detect cancer-associated hyperploidy, owing to a constitutively increased exposure of the endoplasmic reticulum (ER)-sessile protein calreticulin (CRT) on the plasma membrane of hyperploid tumor cells.

For decades, tumors have been considered as strictly non-immunogenic entities. Such an over-simplistic view has been definitively abandoned in the 2000s, along with the expanding consensus on Polly Matzinger's “danger theory”. According to this model, the immune system does not simply discriminate between self and non-self entities but rather reacts to situations of danger, irrespective of their origin [5]. In the subsequent years, a consistent amount of preclinical and clinical evidence has been gathered in support of the notion that malignant cells can be recognized and even eliminated by the immune system, at least in the initial stages of oncogenesis [6]. Thus, also cell-extrinsic barriers to oncogenesis exist, most of which are mediated by the immune system. In this setting, multiple mechanisms may explain why developing malignancies may break immune tolerance (Figure 1). First, tumor cells often express tumor-associated antigens that, at least in some circumstances, are able to ignite tumor-specific immune responses. Second, (pre-)malignant cells frequently upregulate plasma membrane receptors that activate cytolytic components of the innate immune system such as natural killer (NK) cells [7]. Third, neoplastic cells treated with some anticancer therapeutics undergo a functionally peculiar type of apoptosis that *de facto* is immunogenic, hence promoting the priming of tumor-specific cytotoxic CD8^+^ cells. Among other processes, the immunogenicity of cell death relies on the exposure of the endoplasmic reticulum (ER)-sessile protein calreticulin (CRT) on cell surface, acting as an “eat-me” signal for antigen-presenting cells that ignite a cognate immune response [8]. We have recently shown that hyperploid cancer cells can also be detected and eliminated by the immune system [9], thus delineating yet another barrier to tumor progression that operates at the interface between cell-intrinsic and cell-extrinsic oncosuppressive mechanisms (Figure 1).

As compared to their parental, near-to-diploid counterparts, hyperploid cancer cells exhibit a constitutively elevated degree of ER stress, manifesting with the inactivating phosphorylation of the eukaryotic initiation factor 2α (eIF2α) and resulting in increased baseline levels of plasma membrane-exposed CRT (ecto-CRT) [9]. At least in part owing to higher amounts of ecto-CRT, hyperploid cancer cells inoculated into immunocompetent hosts form tumors with a reduced incidence as compared to their parental counterparts. Conversely, near-to-diploid and hyperploid cancer cells generate tumors with the same incidence when inoculated into immunocompromised mice [9]. Of note, hyperploid cell-derived tumors recovered from immunocompetent mice exhibit reduced ploidy, lower degrees of ER stress and fewer ecto-CRT than xenografts generated by the same cells in immunodeficient animals [9]. In addition, carcinogen-induced tumors developing in immunocompetent mice exhibit reduced ploidy and lower degrees of ER than the same lesions developing in immunocompromised (*Rag2^−/−^* γc^−/−^, *Stat1^−/−^* or *Dnam1^−/−^*) animals [9]. Overall, our observations indicate that hyperploidy is a cancer-associated trait that is counterselected *in vivo* by the immune system upon the recognition of increased CRT exposure.

To investigate the translational relevance of our findings, we determined nuclear size (as an indicator of ploidy) and eIF2α phosphorylation (as an indicator of ER stress) in breast carcinoma biopsies from patients who either responded or did not respond to neo-adjuvant chemotherapy. In line with previous reports [10], patients that underwent clinical responses to chemotherapy (responders), but not subjects with detectable lesions in spite of six cycles of chemotherapy (non-responders), exhibited high amounts of tumor-infiltrating CD8^+^ T cells over immunosuppressive FOXP3^+^ cells. In addition, the few breast carcinoma cells that could be detected in responders exhibited a decreased nuclear diameter and lower levels of eIF2α phosphorylation as compared to the neoplastic cells of non-responders [9]. These observations indicate that the clinically-relevant activation of the immune system results in the elimination of hyperploid cancer cells, which - conversely - can grow unrestrained in the absence of an adequate immune response.

Our findings reveal an unsuspected mechanism of anticancer immunosurveillance whereby hyperploid malignant cells are recognized and eliminated by the immune system as they exposed increased amounts of CRT on their plasma membrane. Future work will have to establish whether is mechanism constitutes a meaningful target for the development of novel immunotherapeutic strategies against malignancy.
